# Giant Cell Arteritis With Chronic Bronchitis Successfully Treated With Tocilizumab

**DOI:** 10.7759/cureus.40146

**Published:** 2023-06-08

**Authors:** Ryuichi Ohta, Nozomi Nishikura, Hirotaka Ikeda, Chiaki Sano

**Affiliations:** 1 Community Care, Unnan City Hospital, Unnan, JPN; 2 Community Medicine Management, Shimane University Faculty of Medicine, Izumo, JPN

**Keywords:** tocilizumab, older patients, general medicine, rural hospital, chronic bronchitis, giant cell arteritis

## Abstract

Giant cell arteritis (GCA) causes systemic symptoms; however, involvement of the lungs is relatively rare compared to other rheumatic diseases such as rheumatoid arthritis and systemic sclerosis. Diagnosis and treatment of GCA complicated by chronic lung diseases can be challenging. In this case, an 87-year-old male presented with the chief complaints of systemic muscular pain and cough. The patient was eventually diagnosed with GCA complicated by chronic bronchitis. Although GCA treatment with chronic bronchitis is uncertain, we treated the patient with tapering doses of prednisolone and tocilizumab, which were effective. In older patients with systemic muscular pain and cough, GCA can be considered a differential diagnosis, and tocilizumab can be a reliable treatment in cases complicated by lung diseases, similar to other rheumatic diseases.

## Introduction

Giant cell arteritis (GCA) causes systemic symptoms; however, the involvement of the lungs is relatively rare compared to other rheumatic diseases, such as rheumatoid arthritis and systemic sclerosis [1,2]. Patients with GCA initially present with a high fever and systemic pain, similar to polymyalgia rheumatica [3]. GCA is complicated by intra- and extracranial artery symptoms, such as transient visual loss, headache, and jaw claudication [3]. Because GCA inflammation focuses mainly on the large vessels, lung, and kidney involvement is not typical. Unlike the high lung involvement rates in systemic sclerosis (60%-70%) and rheumatoid arthritis (10%-20%) [1,2], lung involvement in GCA has not been reported scientifically [4].

Diagnosis and treatment of GCA can be challenging if complicated by chronic lung disease. GCA develops primarily in older adults and is complicated by chronic diseases caused by aging [5]. Chronic bronchitis is also common among older patients, owing to aging and long-term exposure to tobacco and other irritants. Chronic lung diseases do not have effective treatments and can only be treated symptomatically [6]. An 88-year-old male presented with chief complaints of systemic muscular pain, chronic cough, and fever for two months. The patient was eventually diagnosed with GCA complicated by chronic bronchitis and treated with prednisolone and tocilizumab. The prednisolone dosage was tapered, and the patient’s systemic pain and lung condition resolved. Based on this case, we discuss the relationship between GCA and chronic bronchitis and the effectiveness of tocilizumab in treating chronic bronchitis accompanied by GCA.

## Case presentation

An 88-year-old man was admitted to a rural community hospital for two months with chief complaints of systemic muscular pain, chronic cough, and fever. The patient exercised regularly, but two months before admission, he began to experience a dry cough, mainly at night. A few days after the onset of the cough, the patient experienced systemic pain in both shoulders and hips. He had considerable difficulty standing up in the morning. One week before admission, his temperature increased to 38°C at night. The cough and systemic pain worsened simultaneously, and he visited the hospital. He reported no night sweats, weight loss, chills, transient visual loss, headaches, or jaw claudication. He had no history of contact with others who were sick or had experienced trauma, vaccination, or travel. His medical history included hypertension and dyslipidemia, for which he was administered amlodipine (10 mg) and atorvastatin (10 mg) daily to treat these diseases for 10 years.

His vital signs at the visit were as follows: blood pressure, 100/51 mmHg; pulse rate, 89 beats/min; body temperature, 37.0°C; respiratory rate, 18 breaths/min; and oxygen saturation, 91% on room air. The patient was alert to time, place, and person. Physical examination revealed warmth and swelling in both hands, elbows, shoulders, and the left first finger metatarsophalangeal joints. Physical examination revealed tenderness in both the subacromial and lateral femoral bursae. Late crackles were heard at both lung bases. No new heart murmurs were heard, and no other abnormal neurological findings were noted. No apparent abdominal abnormalities or skin eruptions were observed. Laboratory tests revealed inflammatory conditions with high C-reactive protein levels and erythrocyte sedimentation rates (Table 1).

**Table 1:** Initial laboratory data eGFR: estimated glomerular filtration rate; CK: creatine kinase; CRP: C-reactive protein; Ig, immunoglobulin; HCV: hepatitis C virus; SARS-CoV-2: severe acute respiratory syndrome coronavirus 2; HIV: human immunodeficiency virus; HBs: hepatitis B surface antigen; HBc: hepatitis B core antigen; MPO-ANCA: myeloperoxidase anti-neutrophil plasmatic antibody; PR3-ANCA: proteinase 3 anti-neutrophil plasmatic antibody; SS: Sjögren's syndrome; CCP: cyclic citrullinated peptide; S/CO: sample/cut off

Marker	Level	Reference
White blood cells	9.0	3.5–9.1 × 10^3^/μL
Neutrophils	95.4	44.0–72.0%
Lymphocytes	2.5	18.0–59.0%
Monocytes	1.6	0.0–12.0%
Eosinophils	0.1	0.0–10.0%
Basophils	0.4	0.0–3.0%
Red blood cells	4.13	3.76–5.50 × 10^6^/μL
Hemoglobin	12.4	11.3–15.2 g/dL
Hematocrit	36.4	33.4–44.9%
Mean corpuscular volume	88.2	79.0–100.0 fl
Platelets	15.7	13.0–36.9 × 10^4^/μL
Erythrocyte sedimentation rate	19	2–10 mm/hour
Total protein	6.2	6.5–8.3 g/dL
Albumin	3.0	3.8–5.3 g/dL
Total bilirubin	0.8	0.2–1.2 mg/dL
Aspartate aminotransferase	23	8–38 IU/L
Alanine aminotransferase	8	4–43 IU/L
Alkaline phosphatase	82	106–322 U/L
γ-Glutamyl transpeptidase	59	<48 IU/L
Lactate dehydrogenase	146	121–245 U/L
Blood urea nitrogen	16.4	8–20 mg/dL
Creatinine	0.78	0.40–1.10 mg/dL
eGFR	70.6	>60.0 mL/min/L
Serum Na	129	135–150 mEq/L
Serum K	4.4	3.5–5.3 mEq/L
Serum Cl	95	98–110 mEq/L
Serum Ca	8.6	8.8–10.2 mg/dL
Serum P	2.9	2.7–4.6 mg/dL
Serum Mg	1.7	1.8–2.3 mg/dL
CK	39	56–244 U/L
CRP	8.78	<0.30 mg/dL
IgG	834	870–1700 mg/dL
IgM	102	35–220 mg/dL
IgA	462	110–410 mg/dL
IgE	191	<173 mg/dL
KL-6	197	105.3-401.2 U/mL
HBs antigen	0.0	IU/mL
HBs antibody	0.00	mIU/mL
HBc antibody	0.00	S/CO
HCV antibody	0.00	S/CO
Syphilis treponema antibody	0.00	S/CO
SARS-CoV-2 antigen	Negative	Negative
anti-nuclear antibody	40	<40
MPO-ANCA	<1.0	<3.5 U/mL
PR3-ANCA	<1.0	<3.5 U/mL
anti-SS-A/Ro antibody	<1.0	<10.0 U/mL
anti-CCP antibody	<0.6	<5 U/mL
Urine test		
Leukocyte	Negative	Negative
Nitrite	Negative	Negative
Protein	Negative	Negative
Glucose	Negative	Negative
Urobilinogen	Normal	
Bilirubin	Negative	Negative
Ketone	Negative	Negative
Blood	Negative	Negative
pH	6.5	
Specific gravity	1.024	

**Figure 1:**
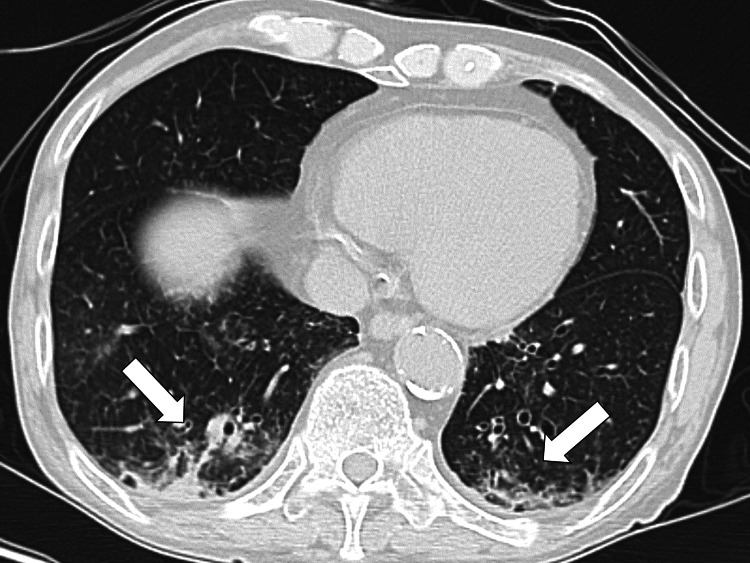
Chest computed tomography showed bilateral interstitial infiltration surrounding the bronchi in the lungs, indicating chronic bronchitis (white arrows).

Autoimmunity test results were negative, including those for rheumatoid factor, anticitrullinated protein antibodies, antinuclear antibodies, and antineutrophil cytoplasmic antibodies. Blood, sputum, and urine cultures (including the tests for tuberculosis) were obtained on admission, and the results were negative after three days. Chest radiography and computed tomography revealed bilateral interstitial infiltration surrounding the bronchi, indicating chronic bronchitis (Figure 1).

Based on the clinical findings, the patient was diagnosed with polymyalgia rheumatica, or seronegative rheumatoid arthritis, and was treated with prednisolone (15 mg/day).

**Figure 2:**
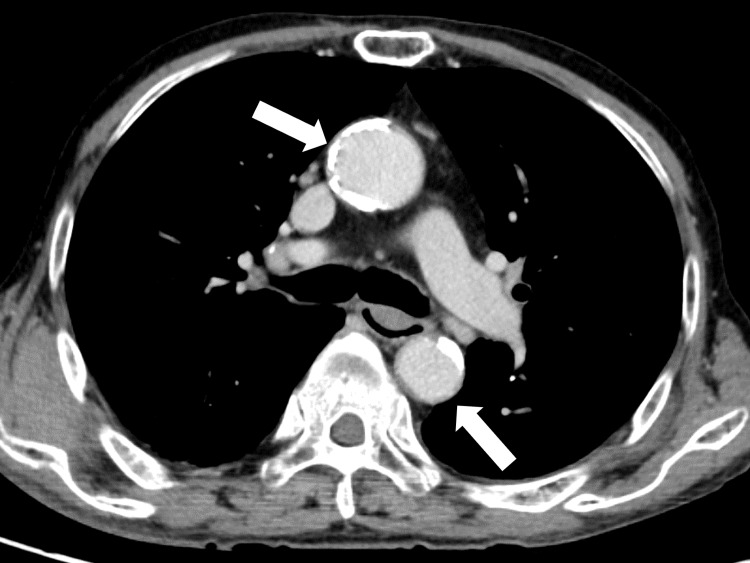
Enhanced chest-to-pelvic computed tomography shows an enhanced and thickened chest aortic wall (white arrows).

On day five after admission, the patient’s symptoms of systemic muscular pain and cough were partially alleviated, but a fever of 38°C and a dry cough at night continued. As a further investigation, enhanced chest-to-pelvic computed tomography was performed, confirming bilateral lung infiltration and enhanced and thickened chest aortic walls (Figure 2).

Physical examination revealed tenderness in both temporal arteries, confirmed by haro signs of the temporal arteries with ultrasound. Based on these findings, the patient was diagnosed with GCA complicated by chronic bronchitis. Prednisolone 60 mg daily was commenced. On day 10, the fever and systemic pain resolved. On day 15, the patient was discharged with an intermittent dry cough caused by chronic bronchitis. In the outpatient department, his cough and systemic pain reappeared when the prednisolone dosage was tapered to 15 mg daily; therefore, tocilizumab (162 mg) every two weeks was started for steroid sparing. Two weeks after starting tocilizumab treatment, the dry cough improved. Within one year of treatment with tocilizumab, the patient’s dry cough disappeared, and a chest CT scan showed no infiltration surrounding the bronchi in the lungs.

## Discussion

This case report confirms the possible relationship between GCA and chronic bronchitis and the effectiveness of tocilizumab in treating chronic bronchitis accompanied by GCA. Multimorbidity is an issue in older patients because multiple diseases coexist or are interconnected. Therefore, treating multiple conditions with a single treatment can be beneficial for avoiding polypharmacy, and physicians should consider the interrelationship between multiple diseases in older patients.

A possible relationship between GCA and chronic bronchitis should also be considered in older patients. In our case, the patient had a chronic cough and was successfully treated with immunosuppression. A previous report has shown that 13% of patients with GCA may exhibit cough symptoms, primarily dry cough [6]. Several previous studies have reported GCA complicated by chronic bronchitis [7]. Chronic bronchitis can be prevalent among older patients; 13% of women and 18.6% of men in older populations have chronic bronchitis diagnosed based on clinical symptoms and computed tomography [8]. The coexistence of chronic bronchitis and GCA, or chronic bronchitis triggered by GCA, can be challenging to differentiate in clinical situations. To effectively manage such situations, assessing responses to treatments for both diseases is a reasonable strategy [9]. Considering the level of emergency, GCA should be initially treated with steroids and other immunosuppressants such as methotrexate and tocilizumab [10]. In addition, considering the exacerbation of lung conditions caused by methotrexate, tocilizumab is preferred for patients with chronic bronchitis and GCA after ruling out occult tuberculosis.

Tocilizumab for chronic bronchitis, accompanied by GCA, can effectively alleviate the symptoms of chronic bronchitis and improve radiological findings. In this case, a patient with chronic bronchitis accompanied by GCA was treated with tocilizumab, and the symptoms and radiological findings disappeared. Treatments for lung involvement in rheumatic diseases are inconclusive [11]. In rheumatoid arthritis, tocilizumab and abatacept are potential candidates for treating interstitial lung disease [12]. However, some reports have shown that tocilizumab may deteriorate lung conditions in rheumatic diseases [13]. Nevertheless, differentiating between exacerbations of rheumatic diseases and lung involvement is challenging. Regarding pathophysiology, inflammation in GCA is induced mainly by an increase in interleukin (IL)-6 in the serum through innate and acquired immunities [5]. When chronic bronchitis presents with CGCA, the pathophysiology can be the same as GCA regarding IL-6 [4]. This case indicates that suppressing IL-6 in the serum can effectively treat chronic bronchitis accompanied by GCA. Tocilizumab is a vital choice for the effective treatment of GCA. When accompanied by chronic bronchitis, tocilizumab can effectively treat inflammation in the arteries and lungs. 

Most patients with GCA are older and have multiple diseases associated with polypharmacy. Treatment of GCA, including chronic bronchitis, based on pathophysiology may reduce redundant medications [10]. When treating rheumatic diseases in older people, the relationship between symptoms should be considered, and appropriate medications based on pathophysiology are crucial. Older patients with multimorbidity and polypharmacy can develop complications due to the disease's interactions. Especially among older patients with autoimmune diseases, clarification of the interactions between the diseases can reduce their medications. As systemic-specific specialists, family physicians should manage complicated older patients comprehensively for a better quality of life among older patients with autoimmune diseases [14,15].

## Conclusions

A possible relationship may exist between GCA and chronic bronchitis, and tocilizumab may be effective for treating chronic bronchitis associated with GCA. Multimorbidity is an issue in older patients, as multiple diseases coexist and may be interconnected. Treating multiple conditions with a single medication may be beneficial for avoiding polypharmacy, and physicians should always consider the possible interrelationships of multiple diseases in older patients.
